# Centralization as the key survival benefit in acute neonatal surgery

**DOI:** 10.3389/fped.2024.1382000

**Published:** 2024-03-14

**Authors:** Manuel Besendörfer, Simone Günster, Katja Linz, Heiko Martin Reutter, Sonja Diez

**Affiliations:** ^1^ Friedrich-Alexander-Universität Erlangen-Nürnberg (FAU), Pediatric Surgery, University Hospital Erlangen, Erlangen, Germany; ^2^ Friedrich-Alexander-Universität Erlangen-Nürnberg (FAU), Hospital for Children and Adolescents, Neonatology and Pediatric Intensive Care, University Hospital Erlangen, Erlangen, Germany

**Keywords:** perinatal center, centralization, neonatal surgery, German medical system, neonatal emergency

## Abstract

**Introduction:**

Centralization of neonatal surgical care for congenital malformations is already under discussion. Acute care of neonatal emergencies in perinatal centers with affiliated hospitals is not uniformly regulated in Germany.

**Materials and methods:**

Analyses are based on acute pediatric surgical care at four affiliated hospitals of a perinatal center. Epidemiologic data and outcome parameters “survival”, “intracerebral hemorrhage”, and “revision of surgical indication” are assessed. Comparison is made between patients receiving surgical treatment at affiliated hospitals (group A) and patients with transfer to the university center for therapy in case of surgical indication for gastrointestinal diseases (group B).

**Results:**

17 group A-patients are compared to 40 group B-patients. Comparison of epidemiological data reveals no significant differences. There is a survival advantage with transfer to the university center (mortality of 29% in group A vs. 2% in group B, *p* = 0.007). Intracerebral hemorrhage occurred more frequently in externally treated patients (group A 24% vs. group B 2%, *p* = 0.024). Surgical indication was revised in 30% of group B at the university center (*p* = 0.011) with consecutive successful conservative treatment.

**Conclusion:**

Transfer of patients at the beginning of the acute phase of gastrointestinal diseases is key to optimize the quality of neonatal surgical care. However, larger population studies should confirm the presented results, discuss restricting factors of real care structures and should rule out bias in triage of patients.

## 1 Introduction

Since 2005, neonatal management structures have been a focal point in the centralization debate in Germany (1). The classification of centers into perinatal centers level I&II and hospitals with perinatal focus, has allowed for the introduction of quality standards to ensure survival and prevent complications. All suspected congenital malformations in neonates are treated in a “Perinatal Center Level I” (PNZ I), which is equipped to handle patients weighing <1,250 g at birth and born <29 weeks of gestational age. In contrast, level II centers are only able to treat preterm neonates weighing >1,250 g at birth and >29 weeks of gestational age, while hospitals with perinatal focus treat neonatal patients weighing >1,500 g at birth and born >32 weeks of gestational age. Risk-adjusted case numbers and survival rates have already been implementing principles of the intended transparency laws for several years. In 2019, The German Gemeinsamer Bundesausschluss (G-BA) established further guidelines for 2025, requiring a minimum of 25 cases per year per center for the treatment of premature infants weighing under 1,250 g birth. This will result in tremendous changes in the neonatal care structures—regardless of conditions for pediatric surgical care.

The presence of a specialized pediatric surgeon available 365 days a year is decisive for achieving the designation of “Perinatal Center Level I or II” (PNZ I/II) and its resulting financial benefits. Therefore, cooperations between smaller pediatric hospitals and pediatric surgeries have been established in many regions (2). This is exemplified in neonatal care of Northern Franconia, where four children's hospitals with neonatal intensive care units have joined together to form a level I perinatal center. Pediatric surgical care is provided by the university center, which also oversees the care for 2,575 births/year (2022) at the affiliated hospitals, in addition to the 1,343 births/year (2022) at the university hospital. On a national level, this high case load is additionally emphasized by 137 German pediatric surgery departments in 2022, providing care for 167 PNZ level I and 45 PNZ level II (3).

Centralization of pediatric surgery has not been as structured so far compared to neonatological dimensions. While discussions have been ongoing for 2–3 decades (4), centralization in Germany currently only includes diagnoses of biliary atresia, bladder exstrophy, and epispadias (2), for which the German Society for Pediatric Surgery provides a structured and multidisciplinary care. Northern European countries have taken a pioneering role of centralization in pediatric surgery due to geographical factors (5) such as low population density, uneven distribution over large areas, and a limited number of children's hospitals. This early realization has led to an increase in caseloads and improvements in all aspects of patient care. After several years, this has resulted in a significant improvement of outcome (6–8), as seen in a Finnish study on biliary atresia. This study confirmed increased rates of clearance of jaundice from 27% to 75% (*p* = 0.001), 2-year jaundice-free native liver survival from 25% to 75% (*p* = 0.002), transplant-free survival from 27% to 75% (*p* = 0.005), and overall survival from 64% to 92% (*p* = 0.082) (7). However, the management of emergency surgical conditions in neonatal care, whether due to congenital or acquired conditions, has not been discussed to our knowledge and faces the challenge of patients with cardiorespiratory instability requiring local surgical treatment.

In summary, this study clarifies the basic structures of German treatment contracts and the organizational process of neonatal surgical care structures in Northern Franconia. In addition, the outcome of neonatal surgical care is evaluated in comparison based on congenital or acquired gastrointestinal diseases with surgical indication to enable an assessment of pediatric surgical centralization.

## 2 Materials and methods

### 2.1 Design of pediatric surgical care at the perinatal center

While preliminary work by the university-based center began in 2011, work has been underway since 2017 on the contractual basis for the structure of pediatric surgical care at the affiliated hospitals. These determine emergency pediatric surgical care for 365 days a year by a specialized pediatric surgeon. If the child can be transferred, the transfer is conducted to the university center; otherwise, emergency surgical care is also possible locally, especially for neonatal interventions. For this purpose, quality assurance structures have been established in the affiliated hospitals. They include

• The provision of facilities suitable for neonatal surgery (in the operating room, at the neonatal intensive care unit)
• The availability of specialized surgical instruments
• The support of operating room nurses and pediatric anesthesiologists, with required both anesthesiological and neonatal care of the patient's anesthesia in emergencies
• Optimized standards procedures, which specify professional, hygienic and patient safety aspects.

In the case of surgeries at affiliated hospitals, the medical care of the university center must also be guaranteed by a second pediatric surgeon, as must be the physician's safety (e.g., in dangerous weather conditions, etc.).

### 2.2 Process of triaging patients and transferring them to the university center

On the basis of the treatment contracts, consultative presentations of affected neonates are made by the affiliated hospital. Bedside visits are carried out promptly by the pediatric surgeons in the affiliated hospitals. By interdisciplinary consensus, the neonate is triaged into “transferable patient” and “non-transferable patient” in case of diagnosis of an emergency gastrointestinal disease with surgical indication.

In case of transferability, the hemodynamic stability of the patient, also with regard to gestational age, current body weight and comorbidities, allows transfer to the university center. The transfer is carried out with the medical support of a specialist from the affiliated hospital. At the university center, complete imaging and laboratory diagnostics are repeated to confirm the diagnosis. The consensus of the interdisciplinary case discussion validates and finally confirms or revises the surgical indication. After surgery, postoperative care is initially continued at the center. After an uneventful postoperative course, transfer back to the affiliated hospital for further treatment is planned at an early stage.

In the case of a patient who cannot be transferred, the affiliated hospital has to decide carefully and individually. The risks of cardiorespiratory instability and immaturity of the child are weighted higher by the attending physicians than the advantages of a transfer to the center. The indication for surgical treatment is therefore made on an interdisciplinary basis. The surgery is planned, staffed and performed by the colleagues of the affiliated neonatological intensive care unit together with the surgical coordinator. The surgical team is provided by the university center. After surgical treatment, postoperative care is ensured by the affiliated hospital in combination with regular visits by the surgeon. In case of complex postoperative care or complications, a transfer to the university center is still possible after hemodynamic stabilization of the child.

**Figure 1:**
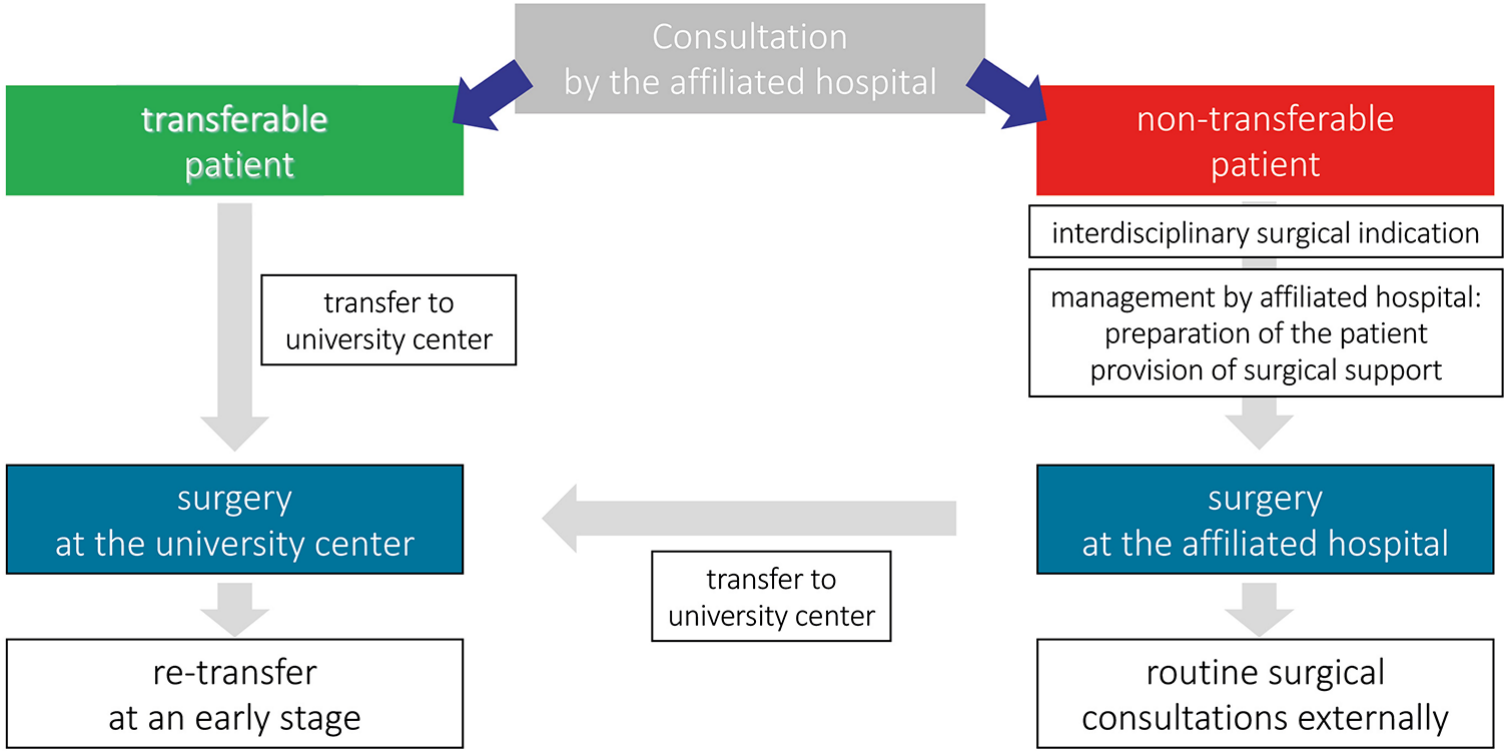
Patients' management of the presented perinatal center regarding neonatal acute abdomen. Classification is made based on the possibility of transfer to the university hospital and an according surgical management is initiated.

An overview of the organizational structure can be found in Figure 1.

### 2.3 Study design and statistical analysis

Following the establishment of the organizational structures, emergency surgical procedures have been performed regularly by the university pediatric surgery at affiliated hospitals of the Perinatal Center since 2018. In the present study, these were analyzed in the period from January 2018 to May 2023. The study was approved by the Ethics Committee of the Friedrich-Alexander-Universität Erlangen-Nürnberg (FAU, No. 23-295-Br) and was conducted in accordance with the Declaration of Helsinki (1964). Patients were considered according to the following inclusion criteria:

• Diagnosis of gastrointestinal disease, congenital or acquired with surgical indication
• Diagnosis within the first month of life
• Birth in one of the affiliated hospitals of the Perinatal Center.

For further analysis, patients were categorized into two groups:

(A) Patients treated exclusively at the affiliated hospitals (group A).
(B) Patients transferred to the university center with surgical indication for further therapy (group B).

Data on demographics as well as clinical course were collected retrospectively from existing medical records. The following parameters were defined as primary outcome variables: survival and occurrence of intracerebral hemorrhage. Reassessment of surgical indication was analyzed as a secondary outcome variable. The clinical assessments of neonates and decisions to transfer were made by interdisciplinary consensus of an in all cases comparable team, as described above.

**Figure 2:**
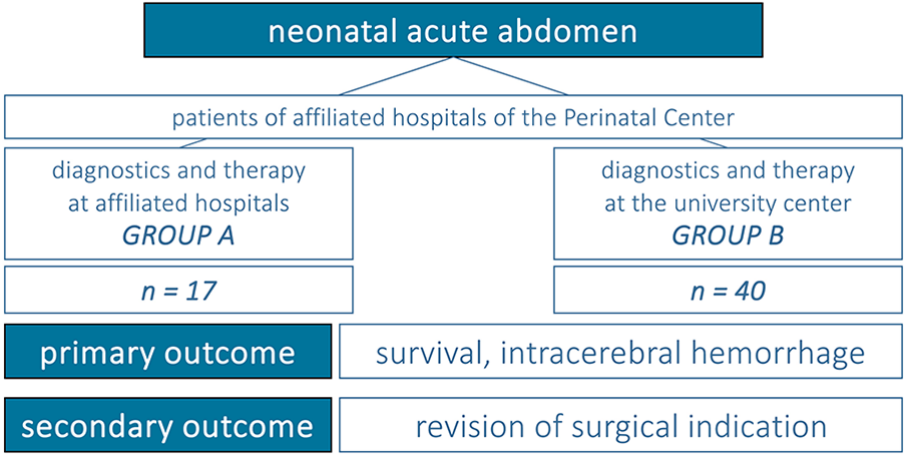
The study's design.

Figure 2 summarizes the study design. Binary data in unpaired samples were compared using the Chi^2^ or Fisher's exact test. Mann–Whitney *U*-test was applied for ordinal, not-normally distributed data. Values of *p* < 0.05 were considered statistically significant.

## 3 Results

### 3.1 Demographic data

During the study period from 2018 to 2023, a total of 87 neonates from the affiliated hospitals were treated by university pediatric surgeons. Diagnosis and surgical treatment were conducted at the affiliated hospital in 21 patients, 66 patients were transferred to the university center for further diagnosis and therapy. In 5/66 patients, surgical treatment had been conducted at the affiliated hospital prior to transfer to the university hospital (8%).

After exclusion of other diagnoses (e.g., congenital malformations, incarcerated inguinal hernia, renal insufficiency for peritoneal dialysis catheterization), patients with emergency gastrointestinal disease and surgical indication could be identified and classified into group A (treated exclusively at the affiliated hospital, *n* = 17, 81%) and group B (treated at the university center, *n *= 40, 61%). Table 1 summarizes demographic data.

**Table 1:** Demographic data of the study's population.

	Group A	Group B	*p*-value
All patients	21	66	
Patients with emergency gastrointestinal disease	17 (81%)	40 (61%)	
Gestational age at birth [Median in weeks (range)]	30.6 (24.0–41.6)	33.9 (23.9–41.7)	0.163
Birth weight [Median in g (range)]	1,615 (490–3,530)	1,770 (480–3,900)	0.397
Patients with very low birth weight [*n* (%)]	8 (47%)	18 (45%)	1.000
Age at transfer to the center/OR [Median in *d* (range)]	6 (0–45)	4 (0–90)	0.674
Diagnoses			
Atresia/intestinal duplication	3 (17.5%)	8 (20%)	0.818
Abdominal wall defect	1 (6%)	3 (7.5%)	
Hirschsprung	2 (12%)	3 (7.5%)	
Meconium ileus	3 (17.5)	6 (15%)	
Necrotizing enterocolitis	8 (47%)	16 (40%)	
Others	0	4 (10%)	

Group A: Patients cared for exclusively at the affiliated hospitals of the Perinatal Center.

Group B: Patients with surgical indication transferred to the university center for further therapy.

### 3.2 Therapy and outcome

Mortality of group B was low with death in one patient due to trisomy 18 (non-survivable chromosomal aberration). Comparatively, an overall survival of 12/17 was observable in group A (71% vs. 98% in group B, *p* = 0.007). In all 5 cases, death was caused by progression of sepsis with multiorgan failure.

Intracerebral hemorrhage occurred before surgery in 4/17 patients of group A (24%) and in 1/40 patients of group B (2%, *p* = 0.024). In the one patient who was transferred to the university center for further therapy, cerebral hemorrhage occurred before transfer to the center and showed no relevant progression after transfer and surgery.

**Figure 3:**
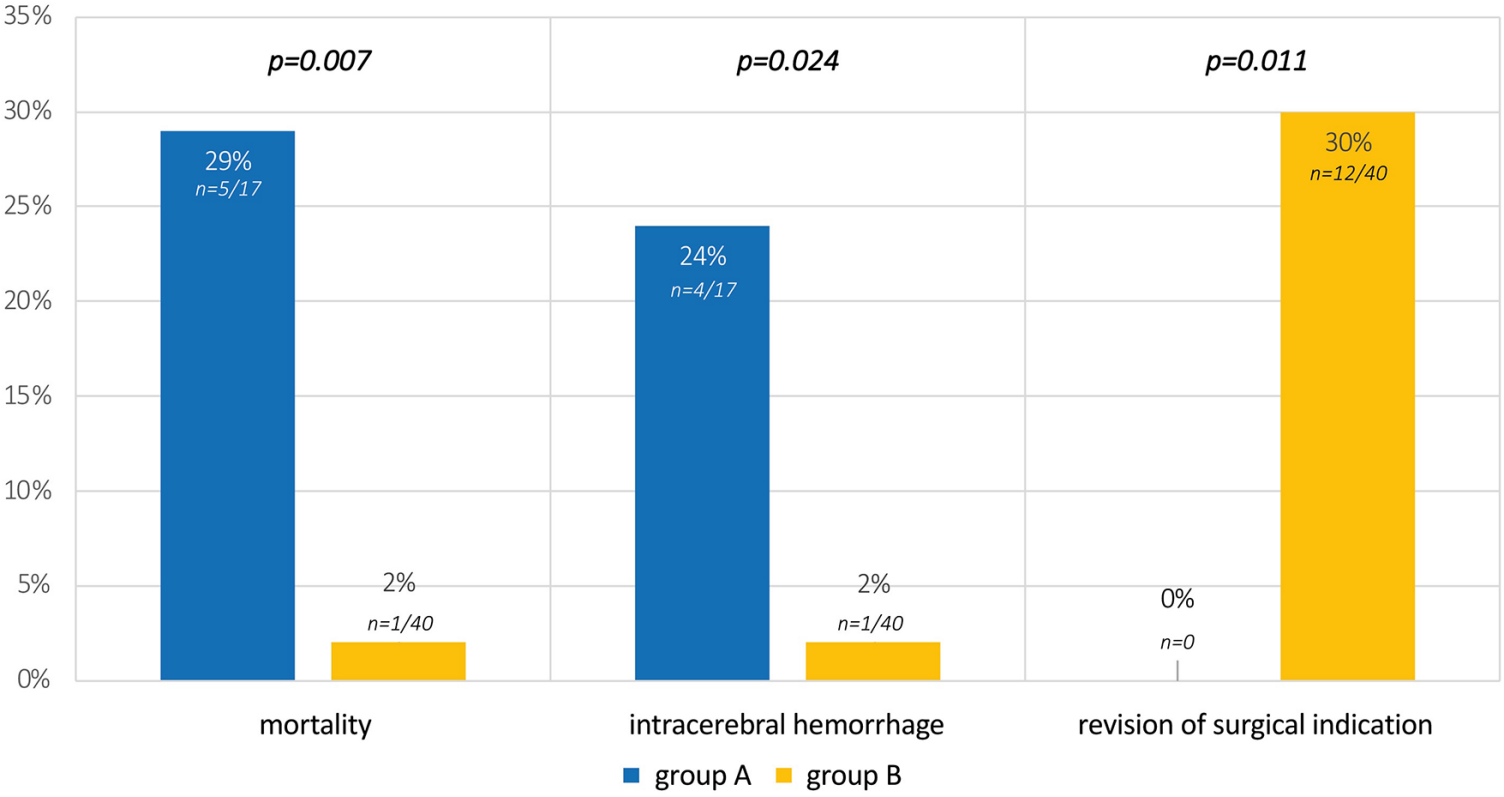
Outcome parameters and results of the study. Group A: patients cared for exclusively at the affiliated hospitals of the Perinatal Center; Group B: patients with surgical indication transferred to the university center for further therapy.

Regarding the secondary outcome parameter, 30% of cases (group B, *n* = 12/40) did not receive surgical treatment after reassessment of the findings at the university hospital, although surgery was originally considered necessary. This is highlighted by 7/16 patients (40%) with diagnosis of necrotizing enterocolitis and an externally set surgical indication (suspected perforation in the imaging diagnostics), whose imaging findings at the center did not confirm the suspected diagnosis and no surgery was performed. Similarly, surgery was avoided in 3/6 patients with meconium ileus (50%) by conservative measurements after transfer to the university center. The comparison of all outcome variables is summarized in Figure 3.

Transfer back from the center to the affiliated hospitals of the Perinatal Center close to home was possible in 14/40 patients of group B (35%), and 24 patients (60%) were discharged directly from the university hospital after consultation with the referring physician.

## 4 Discussion

Pediatric surgeons often face the challenge of performing low volume, high risk interventions, decisively shaping the scope of their quality (2). The pursuit of quality improvement through centralization in this specialty must thus be evaluated with caution.

### 4.1 Multidisciplinarity as the key to success

With regard to this low case volume, multidisciplinarity must be regarded as central to quality assurance in pediatric surgery. Optimal neonatal surgical management is based in equal parts not only on surgical expertise, but also on anesthesiological aspects and perioperative neonatological care. In the acute treatment phase, highly qualified pediatric-radiological knowledge also leads to a survival advantage. Only through cooperation between these four disciplines optimal solutions can be found for the complex neonatal population. Therefore, structural and political decisions for pediatrics and neonatology also remain of great importance for pediatric surgical care.

The centralization principles in the Netherlands can be seen as a pioneer. The guidelines for pediatric centralization were not only made dependent on case numbers. Soft factors such as treatment modalities and local care structures (continuous presence of multidisciplinary teams of sufficient size, good accessibility, and neonatology and pediatric intensive care units) were also considered relevant (9).

Multidisciplinarity could also help to overcome one of the main limitations of the proposed approach: Evidence for a central bias in the assessment of transferability should be taken with caution. Bias could be observed on both sides. Transfer of patients with suspected meconium ileus might be preferred by treating surgeons, anticipating a more successful conservative management at the university center. In contrast critically ill patients are more likely to be treated surgically in the affiliated hospitals, which can have a significant impact on mortality rates. Moreover, financial aspects must be taken into account as influencing factors, as discussed below. We address this limitation of the presented study and advocate for a multidisciplinary decision making that is as objective as possible, at best based on scores and indices of hemodynamic parameters.

### 4.2 Centralized care of the acute phase

The current policy of centralization is in line with the focused goal of pediatric surgical care. Advantages and disadvantages have been carefully assessed and have already been partially implemented in Germany. Aspects of neonatal emergency care were discussed as a secondary issue in this context. For these diagnoses, the merger of smaller hospitals appears to support local care structures with sometimes only one to two individual pediatric surgeons for economic and reputational reasons—a development in which ensuring quality standards and thus the health of the child appears to be of secondary importance.

The results of the present study confirm doubts and disadvantages of these decentralized care structures. Different structural conditions of the affiliated hospitals put pediatric surgical care to the test. The results confirm clear differences in quality, which not only affect the outcome of patients in individual cases, even if we accept the limitations of a small population, single-center study.

Presented results can rule out discussed risk factors of the transfer of acute, critically ill patients (influence of low birth weight, occurrence of intracerebral hemorrhage or hemodynamic instability). In summary, the transfer of the patient in the acute phase from affiliated hospitals to the university center can be considered safe and life-saving, as also shown in a comparable study from the USA (10). Interdisciplinary case discussions at the university center are based on newly gained knowledge and re-evaluate surgical indications; surgeries can be performed in a more targeted manner due to the routinely optimized intensive care. The transfer back to the affiliated hospital, which is planned early in the postsurgical convalescence phase, gives patients and parents the opportunity to receive further care close to home and allows the treating physicians to maintain treatment continuity. In this way, the university center can care for a large number of patients with acute and complex illness. This circle ultimately becomes a circle of life for the individual, but a circle of quality for the entirety of the treated cases in a hospital.

However, the limitations of the proposed concept need to be discussed and should lead to a cautious interpretation of the data. This circle of transfer requires high capacity and flexibility of university centers for the treatment of patients in the emergency setting. In reality, capacity and workload issues might be restricting factors for the individual best care and should certainly be considered politically in the centralization debate. The referral of patients should not be an option to end treatment prematurely at the university center, but should be decided on the basis of interdisciplinary discussion in close cooperation with the transferring, affiliated hospital.

### 4.3 Cost-efficiency in neonatal surgery?

Health policy decisions are highly affected by conflicts of interest, especially when it comes to the transfer of critically ill patients to specialized centers. In this regard, financial aspects influence treatment decisions and have led in many countries to compromises that do not prioritize patient interests (11).

Cost-effectiveness must always take second place to optimal patient care in pediatric surgical care of preterm and term neonates. Not only should the coverage of transport costs for the individual patient be ensured, but fixed payments should also be discussed as financial compensation for hospitals that transfer patients. An early referral of patients to the affiliated hospital might additionally support the cost balancing. Finally, the affiliated hospital must not be disadvantaged by the quality gain resulting from centralization. This and other principles of effective centralization, as discussed by Vonlanthen et al. (12), are crucial instruments for the success of quality improvement.

### 4.4 Basis of future quality optimization

Center transfer in the acute phase must be seen as the optimal way in neonatal emergency care. Nevertheless, advantages can be discussed based on the establishment of neonatal surgery at the affiliated hospitals. Quality assurance is achieved through standardization, good accessibility and regular communication, availability of surgical instruments, and established conditions of triaging. In this regard, the transfer of university principles was kept central and interdisciplinarity was promoted in the given example. The above-mentioned four pillars (pediatric surgery, pediatric anesthesia, pediatric radiology and neonatology/pediatrics) continue to be equally involved in the success of surgery and the individual outcome.

Although the present study provides only a singular insight into neonatal surgical care in Northern Franconia, principles can be applied to other specialties in the process of centralization. However, it is important to note the varying quality standards between smaller hospitals and between university hospitals. In particular, the structural differences in university hospitals, which have different quality standards, should be the focus of assessments in larger population studies. Additionally, the numerous pediatric surgeons treating gastrointestinal congenital and acquired conditions could serve as another basis for centralization in Germany (3).

In summary, treatment of the acute phase of this critically ill population must be reserved for the center. The definition of quality standards, early communication for consultations and good structuring in collaboration with the affiliated hospitals remain essential for quality assurance. In this context, mortality and morbidity review and debriefing conferences with all disciplines have also been established, facilitating close-to-home follow-up and supporting the authorization of the individual clinics in the process of centralization.

## Data Availability

The original contributions presented in the study are included in the article/Supplementary Material, further inquiries can be directed to the corresponding author.
